# Synergistic killing of FLT3ITD-positive AML cells by combined inhibition of tyrosine-kinase activity and N-glycosylation

**DOI:** 10.18632/oncotarget.15772

**Published:** 2017-02-28

**Authors:** Dimitrios Tsitsipatis, Ashok Kumar Jayavelu, Jörg P. Müller, Reinhard Bauer, Dirk Schmidt-Arras, Siavosh Mahboobi, Tina M. Schnöder, Florian Heidel, Frank-D. Böhmer

**Affiliations:** ^1^ Institute of Molecular Cell Biology, CMB, Jena University Hospital, Jena, Germany; ^2^ Institute of Biochemistry, Christian-Albrechts-University Kiel, Kiel, Germany; ^3^ Institute of Pharmacy, Faculty of Chemistry and Pharmacy, University of Regensburg, Regensburg, Germany; ^4^ Innere Medizin II, Hämatologie und Onkologie, Universitätsklinikum Jena, Jena, Germany; ^5^ Leibniz Institute on Aging, Fritz-Lipmann-Institute, Jena, Germany; ^6^ Current address: Institute of Nutrition, Department of Nutrigenomics, Friedrich-Schiller-University, Jena, Germany; ^7^ Current address: Max-Planck Institute of Biochemistry, Department of Proteomics and Signal Transduction, Martinsried, Germany

**Keywords:** acute myeloid leukemia, FLT3ITD, tunicamycin, selective cytotoxicity

## Abstract

Fms-like tyrosine kinase 3 (FLT3) with internal tandem duplications (ITD) is a major oncoprotein in acute myeloid leukemia (AML), and confers an unfavorable prognosis. Interference with FLT3ITD signaling is therefore pursued as a promising therapeutic strategy. In this study we show that abrogation of FLT3ITD glycoprotein maturation using low doses of the N-glycosylation inhibitor tunicamycin has anti-proliferative and pro-apoptotic effects on FLT3ITD-expressing human and murine cell lines. This effect is mediated in part by arresting FLT3ITD in an underglycosylated state and thereby attenuating FLT3ITD-driven AKT and ERK signaling. In addition, tunicamycin caused pronounced endoplasmatic reticulum stress and apoptosis through activation of protein kinase RNA-like endoplasmic reticulum kinase (PERK) and activation of the gene encoding CCAAT-enhancer-binding protein homologous protein (*CHOP*). PERK inhibition with a small molecule attenuated *CHOP* induction and partially rescued cells from apoptosis. Combination of tunicamycin with potent FLT3ITD kinase inhibitors caused synergistic cell killing, which was highly selective for cell lines and primary AML cells expressing FLT3ITD. Although tunicamycin is currently not a clinically applicable drug, we propose that mild inhibition of N-glycosylation may have therapeutic potential in combination with FLT3 kinase inhibitors for FLT3ITD-positive AML.

## INTRODUCTION

Acute myeloid leukemia (AML) is a frequent form of leukemia in adults. Despite significant improvements in the past, treatment possibilities are still limited and prognosis especially for elderly patients is unfavorable [1, 2]. Among the different genetic aberrations associated with AML, mutations in the gene encoding Fms-like tyrosine kinase-3 (FLT3) are frequent [3]. The most prevalent type of mutation – internal tandem duplications (ITD) of sequence in the juxtamembrane or the first kinase domain–leads to strong constitutive activation of the encoded kinase FLT3ITD, and is associated with particularly bad prognosis [1, 4, 5]. Caused by its constitutive kinase activity, the biogenesis of FLT3ITD is impaired and cells harbor a relatively large amount of immature, incompletely glycosylated FLT3ITD protein [6]. Differential analysis of the signaling quality of intracellular retained or surface-membrane located FLT3ITD revealed qualitative differences. Intracellular FLT3ITD, e.g. still bound to the endoplasmic reticulum (ER) efficiently activates STAT5, a hallmark of cell transformation, whereas surface-bound FLT3ITD activates AKT and ERK signaling [7, 8]. Both pools of FLT3ITD thereby cooperate in cell transformation. Drugs which further impair glycosylation of FLT3ITD were found being anti-proliferative for FLT3ITD-positive AML cells. One of these compounds is fluvastatin, a clinically applied inhibitor of mevalonate synthesis, which apart from blocking cholesterol synthesis also inhibits N-glycosylation by depleting cells of dolicholphosphate [9]. The other compound is 2-deoxy-D-glucose. This compound depletes cells of ATP, but also impairs N-glycosylation [10]. A possible reason for selective inhibition of FLT3ITD-positive cells by compounds affecting glycosylation may be a further shift of FLT3ITD towards the intracellular localization, thereby abrogating signaling from the cell surface and in turn cell transformation. Tunicamycin is a bacterial antibiotic, which specifically inhibits the transfer of activated sugars to dolicholphosphate, an essential step in N-glycosylation of proteins at the ER [11, 12]. Because of the fundamental role of this process, tunicamycin has cytotoxic potential, but at low or moderate doses, more specific effects on different cell types have been observed. Tunicamycin has been shown earlier to arrest underglycosylated FLT3ITD in an ER-bound state and to promote STAT5 activation [7]. Moreover, tunicamycin was also shown to reduce surface levels of other oncogenic membrane receptors [13], and combined cytotoxic activity of tunicamycin with different classes of conventional chemotherapeutic agents has been demonstrated. Importantly, despite its cytotoxicity, at least at higher concentrations, effective doses of tunicamycin for experimental tumor therapy were found tolerable in mice *in vivo*, e.g. in xenotransplantation models of hepatocellular carcinoma and glioma [13, 14]. The cytotoxic activity of tunicamycin on cancer cells has also been related to the generation of ER-stress through the “unfolded protein response (UPR)”. ER-stress inducing agents including tunicamycin could be combined with the mTOR inhibitor rapamycin to cause regression of Ras-driven solid tumors *in vivo* [15]. Here we explored the previously not addressed potential of tunicamycin as targeted therapy for FLT3ITD-positive AML. Applied at rather low concentrations, the compound exhibited mild cytostatic and cytotoxic effects on different cell lines. In FLT3ITD-harboring cells, ER-stress through activation of protein kinase RNA-like endoplasmic reticulum kinase (PERK) and *CCAAT-enhancer-binding protein homologous protein* (*CHOP*), as well as inhibition of FLT3ITD glycosylation and thereby attenuation of AKT and ERK activation contributed to growth arrest and apoptosis induction. In combination with FLT3 kinase inhibitors, tunicamycin exhibited a strong and specific synergy in killing of FLT3ITD-expressing cell lines and primary AML cells.

## RESULTS

### Low doses of tunicamycin inhibit FLT3ITD glycosylation and attenuate AKT and ERK activation

**Figure 1:**
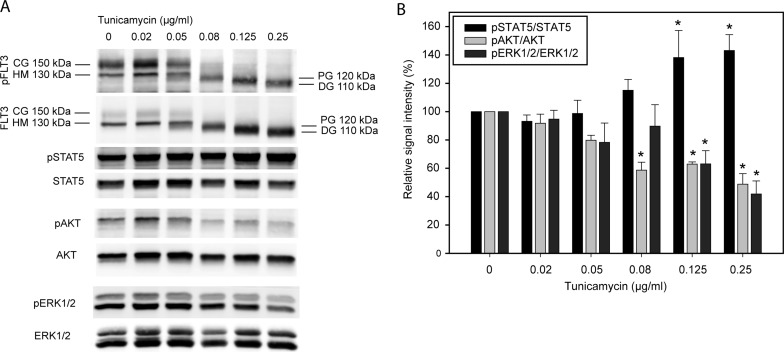
Tunicamycin inhibits glycosylation and modulates signal transduction of FLT3ITD MV4-11 cells harboring endogenous FLT3 ITD were treated with the indicated doses of tunicamycin for 24 h. (**A**) Cell lysates were prepared and whole-cell lysates were subjected to SDS-PAGE and immunoblotting with the indicated antibodies. CG, complex glycosylated form; HM, high-mannose form; PG, partially glycosylated; DG, deglycosylated. Assignments of molecular masses were done by comparison with marker proteins, assignment of the forms was based on these masses and previous observations [6]. Elevated pan-FLT3 signals at higher tunicamycin doses may be a consequence of better recognition of less phosphorylated receptor by the FLT3 antibodies. (**B**) Quantification of three independent experiments (mean ± SD; *significant differences from values in the untreated control).

Selective anti-proliferative and cytotoxic effects of compounds which, among other cellular activities, affect protein N-glycosylation for FLT3ITD-expressing cells have previously been reported. We therefore intended to determine if tunicamycin, a compound whose only known activity is the specific and selective inhibition of N-glycosylation [11], may likewise be selectively cytostatic or even cytotoxic for such cells. The potential of tunicymycin for such a therapeutic approach has previously not been assessed. To determine a meaningful dose-range for such experiments, we initially analyzed the effect of tunicamycin on FLT3ITD glycosylation and the induction of ER-stress. We used the human cell line MV4-11 with endogenous biallelic expression of FLT3ITD for these experiments. As shown in Figure 1A, treatment of these cells with doses of tunicamycin as low as 0.05–0.1 μg/ml were sufficient to markedly reduce the formation of fully glycosylated FLT3ITD and led to accumulation of underglycosylated species. While inhibition of glycosylation also caused reduction in the amount of phosphorylated complex glycosylated receptor, phosphorylation of underglycosylated (ER-arrested) FLT3ITD species was reduced but still clearly detectable. FLT3ITD constitutively activates STAT5, AKT and ERK signaling, which can be robustly detected in cell lysates (Figure 1A). As reported earlier (albeit with much higher tunicamycin concentrations) [7], the tunicamycin treatment at the employed low doses significantly attenuated AKT and ERK activation, and enhanced activation of STAT5 (Figure 1B). Similar observations were made when using MOLM13 cells, another human AML cell line with endogenous FLT3ITD expression (Supplementary Figure 1).

### Tunicamycin induces apoptosis through generation of ER-stress

**Figure 2:**
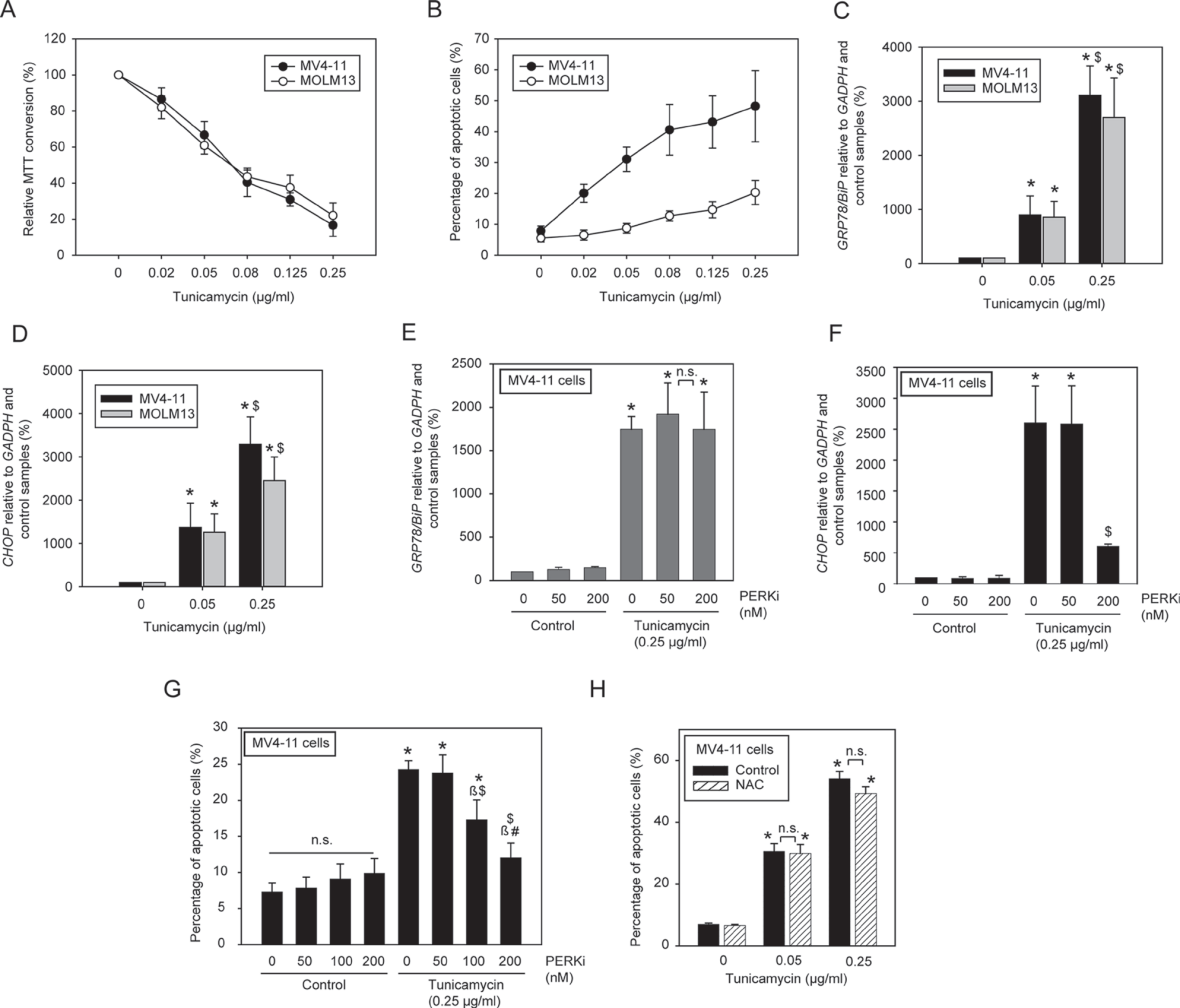
Low doses of tunicamycin induce apoptosis in human AML cell lines harboring FLT3ITD through activation of a PERK/*CHOP* axis MV4-11 cells and MOLM13 cells, respectively, were treated with the indicated concentrations of tunicamycin for 72 h (**A**) or 24 h (**B**). Subsequently the amount of viable cells was measured by MTT conversion (A) or apoptosis was determined using the Annexin V method (B). Data are means ± SD; A, *n* = 4; B, *n* = 3. (**C**, **D**) MV4-11 or MOLM13 cells were treated with the indicated tunicamycin concentrations for 24 h. Subsequently RNA was extracted and mRNA expression of *GRP78/BiP* (C) or CCAAT-enhancer-binding protein homologous protein (*CHOP*) (D) were determined by RT-qPCR. Data are means ± SD; (*n* = 3. *significantly different from untreated controls. $ significant difference 0.05 vs. 0.25 μg/ml tunicamycin). (**E**, **F**) Effect of the PERK inhibitor GSK2606414 (PERKi) on the ER-stress response. Cells were treated with the indicated concentrations of PERKi in absence or presence of tunicamycin for 24 h. Induction of *GRP78/BiP* or *CHOP* mRNA were estimated by RT-qPCR. (**G**) Inhibition of PERK attenuates tunicamycin-evoked apoptosis. Cells treated as in (E, F) with different concentrations of PERKi, were assessed for apoptosis induction by Annexin V staining (means ± SD; *n* = 3; n.s., not significant; significant differences: * vs. untreated control; $ vs. 0 nM PERKi; $ vs. 50 nM PERKi; # vs. 100 nM PERKi). (**H**) ROS quenching by N-acetylcysteine has no influence on tunicamycin-induced apoptosis (means ± SD; *n* = 2; n.s., not significant; *significantly different from untreated control). Note that in different experimental series carried out at different times (e.g. the one in G, and H) the quantitative apoptotic response to tunicamycin at a given concentration varied, possibly related to different tunicamycin batches. Therefore scales in these panels were adjusted to the maximal responses.

Treatment of MV4-11 and MOLM13 cells with tunicamycin alone caused a dose-dependent inhibition of the amount of viable cells as scored by 3-(4, 5-dimethylthiazolyl-2)-2, 5-diphenyltetrazolium bromide (MTT) assays (Figure 2A) and promoted apoptosis detected by Annexin V binding (Figure 2B). While inhibition in MTT assays was similar for both cell lines, apoptosis induction was clearly more pronounced for MV4-11 than for MOLM13 cells, suggesting that tunicamycin is anti-proliferative in both cell lines, but significantly more cytotoxic for MV4-11 cells. Given that FLT3ITD-driven AKT and ERK signaling in both cell lines was only partially inhibited and not abrogated, apoptosis induction appeared to be not exclusively mediated through inhibition of FLT3ITD signal transduction. Similar observations were made in FLT3ITD expressing 32D cells. Tunicamycin reduced cell growth assessed by MTT assays, but the cells were refractory to tunicamycin-induced apoptosis (Supplementary Figure 2). Tunicamycin also promoted apoptosis of cells which do not express FLT3ITD. These included parental 32D cells, and wild-type FLT3-expressing 32D cells, which were analyzed in the presence of IL-3 (Supplementary Figure 3A). Also, viability of U937 cells, which are not transformed by a transmembrane oncoprotein, was inhibited by relatively low concentrations of tunicamycin. In contrast, K562 cells, which are transformed by the cytoplasmic oncogenic tyrosine kinase BCR-ABL, were comparatively resistant to the compound (Supplementary Figure 3B).

As reported earlier, arrest of glycoprotein biogenesis by tunicamycin causes ER-stress and this can translate into cytotoxicity [16, 17]. Indeed, the expression of two marker genes of ER-stress and UPR, *GRP78/BiP* and *CHOP* [18, 19], was greatly enhanced upon treatment with tunicamycin within the dose-range found to be cytotoxic for the FLT3ITD expressing human AML cell lines (Figure 2C, 2D). Similar observations were made in murine 32D cells stably expressing FLT3ITD, except that the tunicamycin concentrations required for ER-stress induction were significantly higher (Supplementary Figure 2A–2C). ER-stress mediated activation of *CHOP* occurs downstream of activated PERK [20]. Recently, potent and selective PERK inhibitors (PERKi) have been developed, including GSK2606414, which has been shown to rescue ER-stress induced apoptosis in neuronal cells *in vitro* and *in vivo* [21]. We employed this compound for assessing the possible contribution of the PERK-*CHOP* pathway to tunicamycin-induced apoptosis in MV4-11 cells. Indeed, GSK2606414 potently inhibited *CHOP* activation in these cells but had no effect on tunicamycin-induced *GRP78/BiP* induction, which occurs downstream of the ER-stress sensing inositol-requiring enzyme 1 (IRE1) [20] (Figure 2E, 2F). Importantly, the PERKi also efficiently attenuated tunicamycin-induced apoptosis in a dose-dependent manner (Figure 2G). This indicates that the PERK/*CHOP* pathway causally contributes to apoptosis induction.

FLT3ITD has previously been reported to cause enhanced formation of reactive-oxygen species (ROS) in AML cells [22–24]. An interplay of ER-stress and ROS formation has likewise been reported [25]. Promoting ROS formation in cancer cells beyond a tolerable threshold has been proposed earlier as a strategy for inducing selective cytotoxicity [26]. We therefore considered the possibility that tunicamycin-mediated ER-stress or FLT3ITD ER-retention may enhance ROS formation beyond such toxic threshold, and in turn cause apoptosis. As reported earlier [23, 24], ROS formation in cells with endogenous FLT3ITD expression such as MV4-11 was readily detected, and the antioxidant N-acetylcysteine (NAC) attenuated ROS formation (Supplementary Figure 4A). However, tunicamycin did not further enhance ROS formation (Supplementary Figure 4B). Consistent with this observation, NAC treatment failed to rescue MV4-11 cells from tunicamycin-induced apoptosis (Figure 2H).

Taken together, attenuation of FLT3ITD-driven AKT and ERK signaling and tunicamycin-induced PERK-*CHOP* activation, but not ROS formation, were implicated in apoptosis induction. Deglycosylation of further yet unidentified glycoproteins may also mediate cytotoxic effects. It can be assumed that differential importance and regulation of these pathways contributes to the very different apoptotic responses of the different analyzed cell lines to tunicamycin exposure.

### Tunicamycin enhances inhibition of cell viability of FLT3ITD expressing cells by FLT3 kinase inhibitors

**Figure 3:**
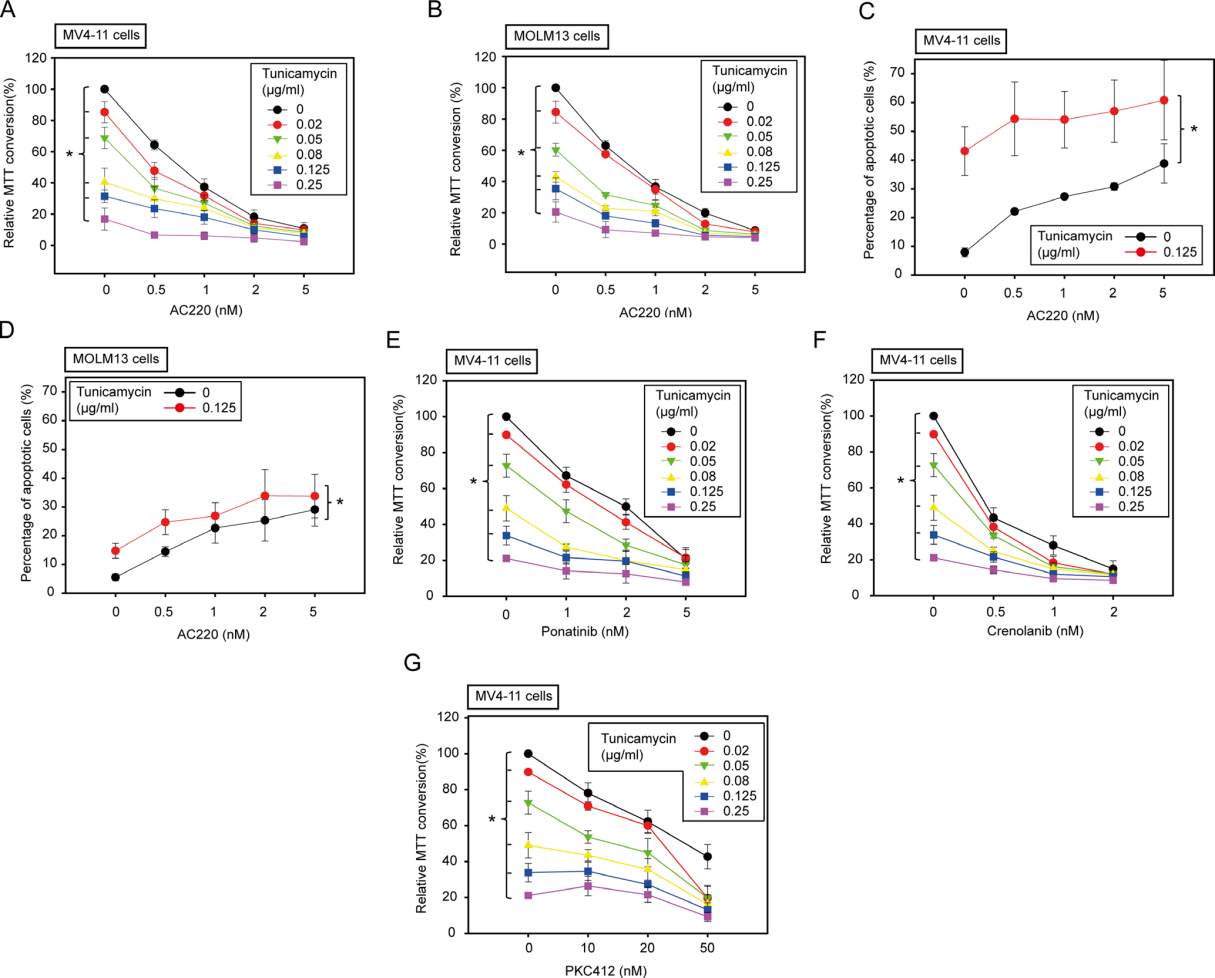
Low doses of tunicamycin enhance the effect of FLT3 kinase inhibitors in human AML cell lines harboring FLT3ITD MV4-11 cells or MOLM13 cells were treated simultaneously with the kinase inhibitors and tunicamycin as indicated for 72 h (**A**, **B**, **E**–**G**) or 24 h (**C**, **D**). Subsequently the amount of viable cells was measured by MTT conversion (A, B, E–G) or apoptosis was determined using the Annexin V method (C, D). Data are means ± SD; *n* = 3. * denotes significant differences between treatment series by two-way ANOVA.

Next, we explored the effect of tunicamycin in combination with the selective FLT3ITD tyrosine kinase inhibitor AC220 (quizartinib), a compound currently in advanced clinical trials for treatment of FLT3ITD positive AML [27]. As reported, AC220 very potently inhibited viability of MV4-11 and MOLM13 cells (Figure 3A, 3B) and promoted apoptosis (Figure 3C, 3D). Importantly, co-incubation with even low concentrations of tunicamycin strongly enhanced AC220-mediated inhibition of cell proliferation (Figure 3A, 3B). Moreover, AC220-evoked apoptosis was also promoted. This effect was more pronounced in MV4-11 than in MOLM13 cells (Figure 3C, 3D). Here even very low tunicamycin concentrations enhanced AC220-induced apoptosis (Supplementary Figure 5). We also assessed the potential effect of AC220 in combination with fluvastatin, a clinically approved mevalonate-synthesis inhibitor, which has been shown to block N-glycosylation of FLT3ITD [9]. Consistent with previously published data, the combination of AC220 and fluvastatin resulted in enhanced inhibition of growth and promotion of apoptosis of MV4-11 cells (Supplementary Figure 6A, 6B). However, the effect of fluvastatin on N-glycosylation in these cells was less pronounced than in other cells [9] (data not shown), suggesting that additional mechanisms may contribute to fluvastatin-induced cytotoxicity in this setting.

We also tested the combination of tunicamycin with other clinically relevant FLT3 inhibitors. We assessed the combined activity with ponatinib, a multikinase type 2-inhibitor [28], with crenolanib, a selective FLT3 type 1-inhibitor [29], and with PKC412 (midostaurin), a FLT3 inhibitor, which will become approved for treatment of FLT3ITD positive AML in the near future [30]. Tunicamycin enhanced the activity of all three compounds as seen by growth inhibition of MV4-11 cells (Figure 3E–3G). These findings indicate that the observed synergism of tunicamycin with FLT3 inhibitors is a more general effect.

When analyzed by the method of Chou-Talalay [31], the combinations of tunicamycin with all tested FLT3 kinase inhibitors were clearly synergistic in certain dose-ranges (Tables 1, 2; Supplementary Tables 1, 2). For example, all tested tunicamycin concentrations synergistically enhanced the inhibitory effect of 2 nM AC220 in MTT assays with MV4-11, MOLM13, and also FLT3ITD-expressing 32D cells (Table 1, Supplementary Table 1), and of 2 nM crenolanib with MV4-11 cells (Table 2). Ponatinib showed synergy with tunicamycin preferentially at 1 and 2 nM, whereas synergy with PKC412 was only seen at 50 nM of this FLT3 inhibitor. Synergy in apoptosis induction with MV4-11 cells was observed in all tested AC220/tunicamycin combinations, but the effect was particularly pronounced at low doses of tunicamycin (Supplementary Table 1).

**Table 1:** Synergistic inhibition of FLT3ITD-expressing human AML cell lines by the combination of the kinase inhibitor AC220 with tunicamycin

Compound treatment		MV4-11 cells	MOLM13 cells
AC220 (nM)	Tunicamycin (μg/ml)	MTT assay (CI)	
0.5	0.02	0.913	1.255
	0.05	0.912	0.713
	0.08	0.941	0.621
	0.125	1.002	0.633
	0.25	0.599	0.528
1	0.02	0.901	1.015
	0.05	0.952	0.828
	0.08	1.009	0.806
	0.125	0.975	0.617
	0.25	0.634	0.495
2	0.02	0.686	0.660
	0.05	0.709	0.512
	0.08	0.748	0.466
	0.125	0.800	0.420
	0.25	0.624	0.456
5	0.02	1.149	1.014
	0.05	1.073	0.876
	0.08	1.035	0.814
	0.125	0.892	0.756
	0.25	0.549	0.745

MV4-11 cells or MOLM13 cells were treated simultaneously with the indicated concentrations of AC220 and tunicamycin. Subsequently the amount of viable cells was measured by MTT conversion after 72 h. Combination indices were determined according to the method of Chou-Talalay [31]. Combinations with synergism (CI < 1) are highlighted grey.

**Table 2:** Synergistic inhibition of MV4-11 cells by the combination of different FLT3 kinase inhibitors with tunicamycin

Compound treatment				MTT assay (CI)		
Tunicamycin (μg/ml)	Ponatinib (nM)	Crenolanib (nM)	PKC412 (nM)	Ponatinib	Crenolanib	PKC412
0.02	1	0.5	10	1.122	0.965	1.121
0.05				1.026	1.013	0.949
0.08				0.715	0.859	0.964
0.125				0.776	0.951	1.049
0.25				0.941	1.054	1.481
0.02	2	1	20	1.004	0.703	1.148
0.05				0.833	0.716	0.942
0.08				0.716	0.759	0.901
0.125				0.894	0.744	0.913
0.25				0.972	0.892	1.295
0.02	5	2	50	1.085	0.832	0.409
0.05				1.011	0.927	0.541
0.08				0.968	0.963	0.544
0.125				0.887	0.998	0.577
0.25				0.916	1.083	0.708

MV4-11 cells were treated simultaneously with the indicated concentrations of the FLT3 kinase inhibitors ponatinib, crenolanib, or PKC412, and tunicamycin. Subsequently the amount of viable cells was measured by MTT conversion after 72 h. Combination indices were determined according to the method of Chou-Talalay [31]. Combinations with synergism (CI < 1) are highlighted grey.

### Specificity of the combined treatment for FLT3ITD expressing cells

**Figure 4:**
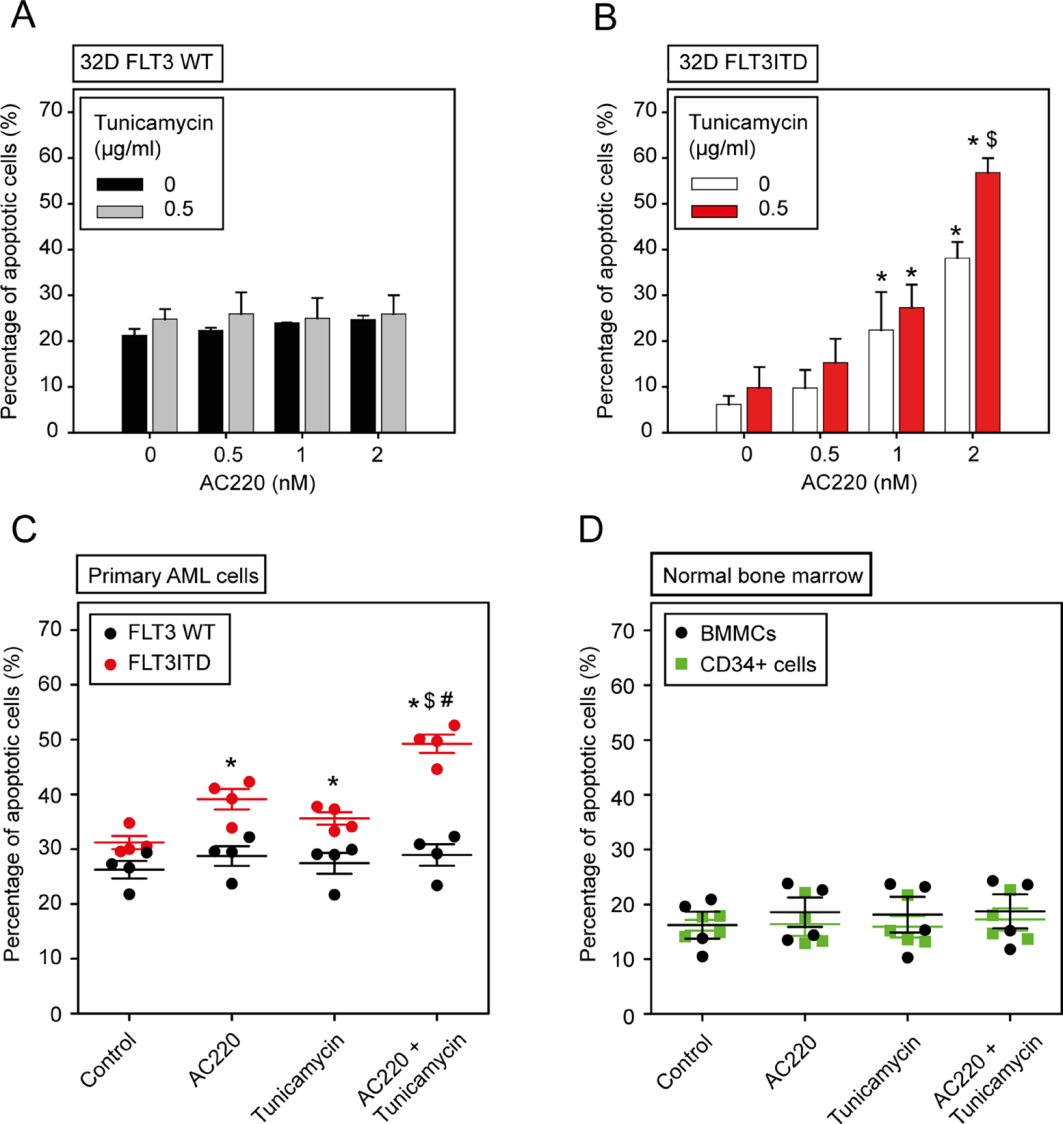
The combination of the kinase inhibitor AC220 and tunicamycin selectively enhances apoptosis of FLT3ITD-expressing cells **(A, B)** 32D cells either expressing wild-type (WT) FLT3 or FLT3ITD were treated simultaneously with the indicated concentrations of AC220 and tunicamycin in absence of IL-3 for 24 h. Subsequently apoptosis was determined using the Annexin V method. Data are means ± SD; *n* = 3; Significances: * different vs. 0 nM AC220; $ different vs. without tunicamycin. (C, D) Selective induction of apoptosis in FLT3ITD-positive primary AML cells. (C) Primary AML cells were isolated from peripheral blood and treated as indicated with AC220 (10 nM), tunicamycin (0.5 μg/ml), or both in cytokine-free medium for 24 h. Apoptosis was measured with the Annexin V method in FLT3/CD135 positive cells. Each dot represents an individual patient sample. Significances: * vs. control; $ vs. AC220 alone; # vs. tunicamycin alone. Note that no significant apoptosis induction was observed in wild-type FLT3 samples. (D) Normal human bone-marrow cells are resistant to FLT3 inhibitor and tunicamycin treatment. Mononuclear cells (BMMCs) were isolated from freshly aspirated normal bone-marrow samples by gradient centrifugation. Cells were incubated in absence of cytokines with the indicated compounds as in (C), and apoptosis was measured with the Annexin V method for total BMMCs, and CD34-positive cells. Each dot represents an individual sample. No significant differences were detected for any treatment.

To explore the specificity of combining tunicamycin and AC220 for cytotoxicity we compared isogenic 32D cells either expressing FLT3ITD or wild-type FLT3. These experiments were performed in the absence of IL-3. As shown in Figure 4, a dose-dependent induction of apoptosis by AC220 in the FLT3ITD-expressing cells was clearly enhanced by adding tunicamycin to the treatment. In contrast, in 32D cells with wild-type FLT3 the basal rate of apoptosis was somewhat higher due to IL-3 starvation, but was not increased by AC220 treatment, neither in absence nor in presence of tunicamycin. Consequently, no combinatorial effect of AC220 was observed in these cells, whereas a clear synergy of tunicamycin with AC220 was detectable in FLT3ITD expressing 32D cells (Table 3). Consistent with these findings, a pronounced synergy of tunicamycin with AC220-mediated inhibition in 32D cells expressing FLT3ITD was also observed in MTT assays (Supplementary Figure 7, Supplementary Table 2).

**Table 3:** The combination of the kinase inhibitor AC220 with tunicamycin synergistically promotes apoptosis of FLT3ITD-expressing but not wild-type FLT3-expressing 32D cells

Compound treatment		32D cells FLT3ITD	32D cells Wild-type FLT3
AC220 (nM)	Tunicamycin (μg/ml)	Annexin V assay (CI)	
0.5	0.5	0.822	0.991
1	0.5	0.796	1.033
2	0.5	0.323	1.038

32D cells either expressing FLT3ITD or wild-type FLT3 cells were treated simultaneously with the indicated concentrations of AC220 and tunicamycin for 24 h. Subsequently apoptosis was determined using the Annexin V method. Combination indices were determined according to the method of Chou-Talalay [31]. Combinations with synergism (CI < 1) are highlighted grey.

We further assessed the effect of the combination of tunicamycin and AC220 on primary human AML cells. In the absence of cytokines cells were treated with vehicle, AC220, tunicamycin, or their combination. Apoptosis was assessed after 24 h of treatment. As shown in Figure 4C, the combination of both agents was selectively cytotoxic for FLT3ITD-positive primary AML cells, but did not affect wild-type FLT3 expressing primary cells. Moreover, normal human bone-marrow derived mononuclear cells (BMMCs) as well as CD34 positive stem- and progenitor cells were resistant to combined tunicamycin and AC220 treatment (Figure 4D).

Taken together, while tunicamycin was not selectively cytotoxic for FLT3ITD-expressing cells as a single compound, it exhibited a pronounced synergy with different clinically relevant FLT3 inhibitors for FLT3ITD-expressing cell lines and primary AML cells. Importantly, cell lines and primary AML cells expressing wild-type FLT3 were resistant to the treatment, as were primary human bone-marrow cells, including CD34-positive progenitor cells.

## DISCUSSION

The main finding of this study is the synergistic enhancement of cytotoxic effects of FLT3ITD kinase inhibition with low doses of tunicamycin in AML cell lines and primary, FLT3ITD-positive AML cells. Tunicamycin is currently not a clinically applicable drug. However, given that tunicamycin at low doses appears to be well tolerated *in vivo* in mouse models [13, 14], and was not markedly cytotoxic to normal human CD34+ bone-marrow cells, our findings suggest that further assessment of FLT3 inhibitor/tunicamycin combinations in preclinical AML models may be warranted.

**Figure 5:**
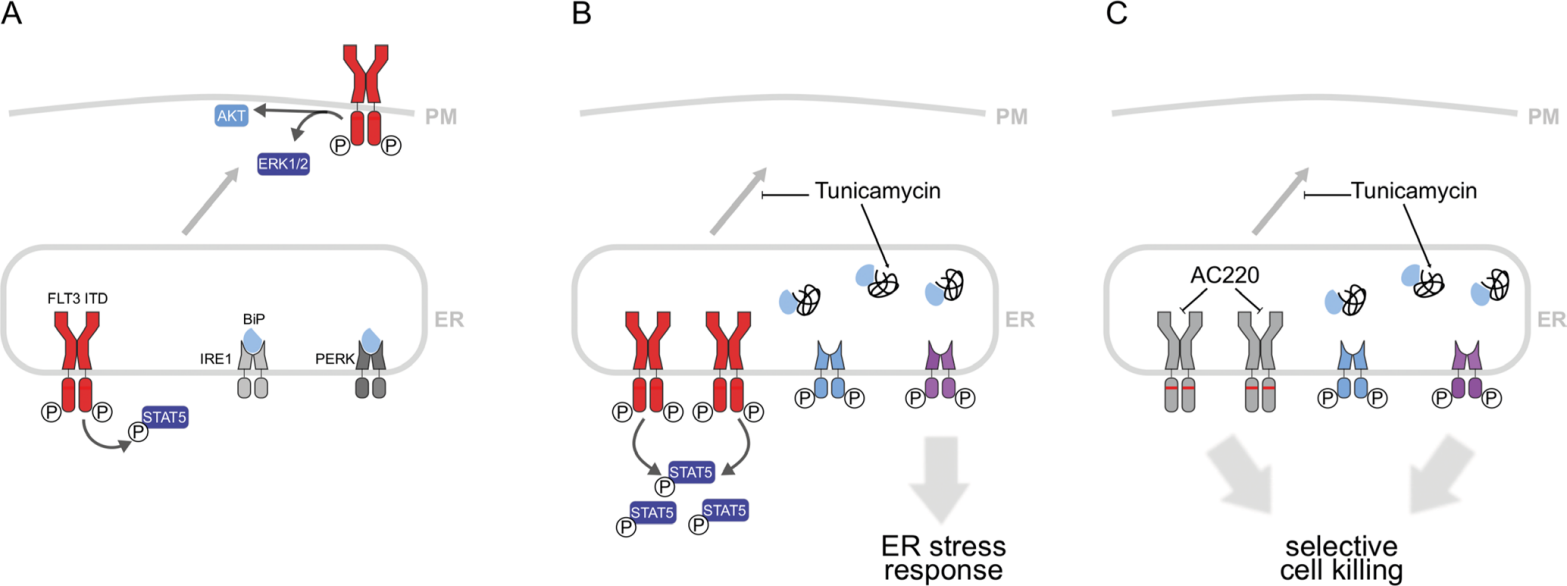
Mechanisms involved in growth inhibition and apoptosis induction by combined treatment of FLT3ITD-positive AML cells with tunicamycin and FLT3 inhibitors **(A)** Two pools of FLT3ITD are important for cell transformation. The pool at the plasma membrane effectively activates ERK and AKT. The intracellular FLT3ITD pool at the ER is the consequence of inefficient biogenesis of FLT3ITD caused by constitutive kinase activity. This pool efficiently activates STAT5. **(B)** Tunicamycin treatment arrests FLT3ITD at the ER in an underglycosylated state. This leads to attenuation of ERK and AKT signaling. Moreover, an ER-stress response is elicited. ER-stress sensing kinases such as inositol-requiring enzyme 1 (IRE1) and protein kinase RNA-like endoplasmic reticulum kinase (PERK) are activated and promote activation of stress response genes, such as *GRP78/BiP* and *CHOP*, respectively (the latter not shown). Inhibition of ERK and AKT activation and induction of ER-stress can mediate growth inhibition and apoptosis. (C) Simultaneous treatment with FLT3 inhibitors and tunicamycin causes selective cell killing by combined abrogation of FLT3ITD kinase signaling, and induction of ER-stress preferentially through PERK activation.

Our results confirm and extend previous findings on the cytotoxic effect of mild inhibition of N-glycosylation for FLT3ITD-transformed cells. Low doses of tunicamycin were shown to efficiently inhibit N-glycosylation of FLT3ITD. In turn, FLT3ITD-driven AKT and ERK activation, both dependent on a fully glycosylated FLT3 receptor at the plasma membrane [7, 8], was attenuated (schematically illustrated in Figure 5). This effect is likely contributing to inhibition of FLT3ITD-mediated transformation and presents a relatively specific mechanism for interference with the activity of this oncoprotein. Enhanced activation of STAT5 in presence of tunicamycin, which was also observed, is not expected to mediate growth inhibition. Indeed, co-treatment of cells with pimozide, a STAT5 inhibiting drug, did not rescue cells from growth arrest and apoptosis induced by tunicamycin (data not shown). Inhibition of N-glycosylation will occur also for other glycoproteins, causing the generation of ER-stress as indicated by the observed induction of ER-stress response genes. Inhibition of PERK attenuated the ER-stress mediated induction of *CHOP*, and partially rescued apoptosis induction by tunicamycin. These data identify the PERK-*CHOP* axis as a relevant pathway for the promotion of apoptosis by tunicamycin in FLT3ITD-positive AML cells (Figure 5). This general mechanism, however, likely also contributes to cytotoxic effects of tunicamycin in cell lines, which do not express FLT3ITD. Importantly, studies of ROS formation and failure of the ROS quencher NAC to attenuate tunicamycin-induced apoptosis render a contribution of ROS unlikely.

Previous reports have suggested a selective cytotoxic potential of inhibiting N-glycosylation for FLT3ITD-expressing cells [9, 10]. The lipid-lowering drug fluvastatin and 2-deoxy-D-glucose were both shown to inhibit N-glycosylation of FLT3ITD and c-Kit mutants and to selectively inhibit growth of FLT3ITD- or mutant c-Kit expressing cells. Treatment with these drugs reduced cell-surface expression of both receptors, which correlated with the cytostatic/cytotoxic response. For fluvastatin, promotion of STAT5 activation but inhibition of ERK and AKT activation was shown and interpreted as consequence of the shift of underglycosylated FLT3ITD/c-Kit to the intracellular pool [9]. Consistent with this earlier report, we observed cytotoxic effects of fluvastatin for FLT3ITD-positive cells, and a strong enhancement of the effect of the FLT3 inhibitor AC220. For the reported effects of 2-deoxy-D-glucose, inhibition of FLT3ITD levels were also observed and attributed to underglycosylation of Sp1, a transcription factor positively regulating c-Kit and FLT3 gene expression [10]. Given the broader cellular effects of both compounds, further mechanisms, in addition to inhibition of N-glycosylation, may contribute to their activity as single agents and their relative selectivity.

According to our data, the specific inhibition of N-glycosylation *per se* by tunicamycin is not sufficiently selective for inhibition of FLT3ITD-mediated cell transformation. However, in conjunction with selective FLT3ITD tyrosine-kinase inhibition, interference with N-glycosylation acts in a synergistic manner and has the potential to serve as a cytotoxic principle for FLT3ITD-positive AML.

## MATERIALS AND METHODS

The human cells lines MV4-11, MOLM13, U937 and K562 were obtained from the Leibniz Institute DSMZ German Collection of Microorganisms and Cell Cultures (Braunschweig, Germany) and cultivated in RMPI1640 medium containing bicarbonate and glutamine (R8758 Sigma-Aldrich, Taufkirchen, Germany), supplemented with 10% heat-inactivated fetal bovine serum (FBS). Parental 32D cells and cell pools transduced with constructs expressing wild-type FLT3 or FLT3ITD [32] were kindly provided by Drs. Hubert Serve (University Hospital Münster, Germany), and Justus Duyster and Rebekka Grundler (Hospital of the Technical University Munich, Germany). They were cultivated in RPMI1640 medium without bicarbonate, buffered with HEPES and containing glutamine (FG1235, Biochrome AG, Berlin, Germany), supplemented with 1 mM sodium pyruvate, 10% heat-inactivated FBS, and 1 ng/ml murine IL-3.

Tunicamycin was obtained from Sigma-Aldrich (Taufkirchen, Germany). AC220 was obtained by chemical synthesis as described [33]. Ponatinib and crenolanib were obtained from Selleckchem (through Biozol, Eching, Germany). PKC412 was from Sigma-Aldrich (Taufkirchen, Germany). All compound treatments were done at a final DMSO concentration of 0.1 %. SDS-PAGE and immunoblotting, and determination of viability using MTT were performed as described earlier [8]. Immunoblots were acquired using an LAS4000 luminescence imager, and quantification of immunoblots was performed using Multi Gauge image software (Fujifilm, Düsseldorf, Germany). Apoptosis was determined by flow cytometry using PE-labeled Annexin V (559763, BD Bioscience Heidelberg, Germany) according to the instructions of the manufacturer.

All work with human primary cells was performed in accordance with the Declaration of Helsinki, and approved by ethic votes of the institutional review board of the University Hospital Jena. Primary AML cells were isolated from peripheral blood as described earlier [24] and cryopreserved at a cell density of 2–5 × 10^7^ cells /ml. The cells were thawed and cultivated in StemSpan^TM^ SFEM II medium (09605 STEMCELL Technologies Cologne, Germany) supplemented with rh-G-CSF rh-SCF, rh-IL-3 and rh-IL-6 (11343133 12343323 11340033 11340063 Immunotools Friesoythe, Germany respectively) for 24 h as described [23]. Thereafter, cells were washed, and treated in the absence of cytokines with the different compounds in StemSpanTM SFEM II medium for 24 h, apoptosis of FLT3-positive cells was determined by flow cytometry using FITC-labeled Annexin V (556420 BD Bioscience Heidelberg, Germany), and PE-labeled human CD135 antibody (558996 BD Bioscience Heidelberg, Germany) according to the instructions of the manufacturer.

Normal human bone marrow mononuclear cells (BMMCs) were obtained by a standard procedure using Ficoll gradient centrifugation (Biocoll S0115, Biochrom AG, Berlin, Germany) from freshly aspirated bone marrow. The cells were washed, seeded and treated with compounds in StemSpan^TM^ SFEM II medium in the absence of cytokines. Apoptosis was determined by flow cytometry using FITC-labeled Annexin V according to the instructions of the manufacturer in both CD34+ and CD34- cells. Gating of CD34+ cells was achieved using PE-labeled human CD34 antibody (343605, Biolegend, San Diego, CA, USA). To assess synergy of drug treatments, the program CalcuSyn (Biosoft, Cambridge, UK) was used. The program calculates the Combination Index (CI) according to the algorithm of Chou-Talalay [31]. A CI < 1 indicates synergy of drug action.

To determine the induction of ER-stress, expression of the genes encoding *BiP* and *CHOP* were determined by RT-qPCR. Isolation of total RNA and cDNA synthesis were performed using the RNeasy Mini Kit (Qiagen, Heidelberg, Germany), and the cDNA synthesis kit of ThermoFisher Scientific (K1612, Schwerte, Germany), respectively, according to the instructions of the manufacturers. A SYBR Green kit was used for qPCR (ThermoFisher Scientific K0221, Schwerte, Germany) with the annealing temperatures adjusted to the different primer pairs: *hBiP* GAAAGAAGGTTACCCATGCAGT (fwd) CAGGCCATAAGCAATAGCAGC (rev) (60°C); *mBip* GGGAAAGAAGGTTACCCATGC (fwd) CGAGTA GATCCACCAACCAGAG (rev) (60°C); *hCHOP* CAGA ACCAGCAGA GGTCACA (fwd) AGCTGTGCCACT TTCCTTTC (rev) (60°C); *mChop* CCCTGCCTTTCA CCTTGG (fwd) CCGCTCGTTCTCCTGCTC (rev) (56°C). As reference genes *hGAPDH* and *mbActin* were also analyzed. The reactions were performed according to the recommendations of the manufacturer.

For signal transduction analysis, 2 × 10^6^ cells per sample were incubated with the tunicamycin concentrations indicated in the figure legends for 24 h. Cell extraction and analysis of signaling by immunoblotting were performed as described earlier [8].

Statistical analyses were performed with one-way or two-way ANOVA as applicable, using the Holm-Sidak method for multiple pairwise comparisons and the program SigmaPlot (Systat Software, Erkrath, Germany).

## SUPPLEMENTARY MATERIALS FIGURES AND TABLES


