# Supplementary material for: Synergistic killing of FLT3ITD-positive AML cells by combined inhibition of tyrosine-kinase activity and N-glycosylation
Source: Oncotarget. 2017 Feb 28;8(16):26613–24. doi: 10.18632/oncotarget.15772 (PMC5432283; doi:10.18632/oncotarget.15772)
Supplement: Supplementary file 1 [file oncotarget-08-26613-s001.pdf]

Synergistic killing of FLT3ITD-positive AML cells by combined inhibition of tyrosine-kinase activity and N-glycosylation

Supplementary Materials

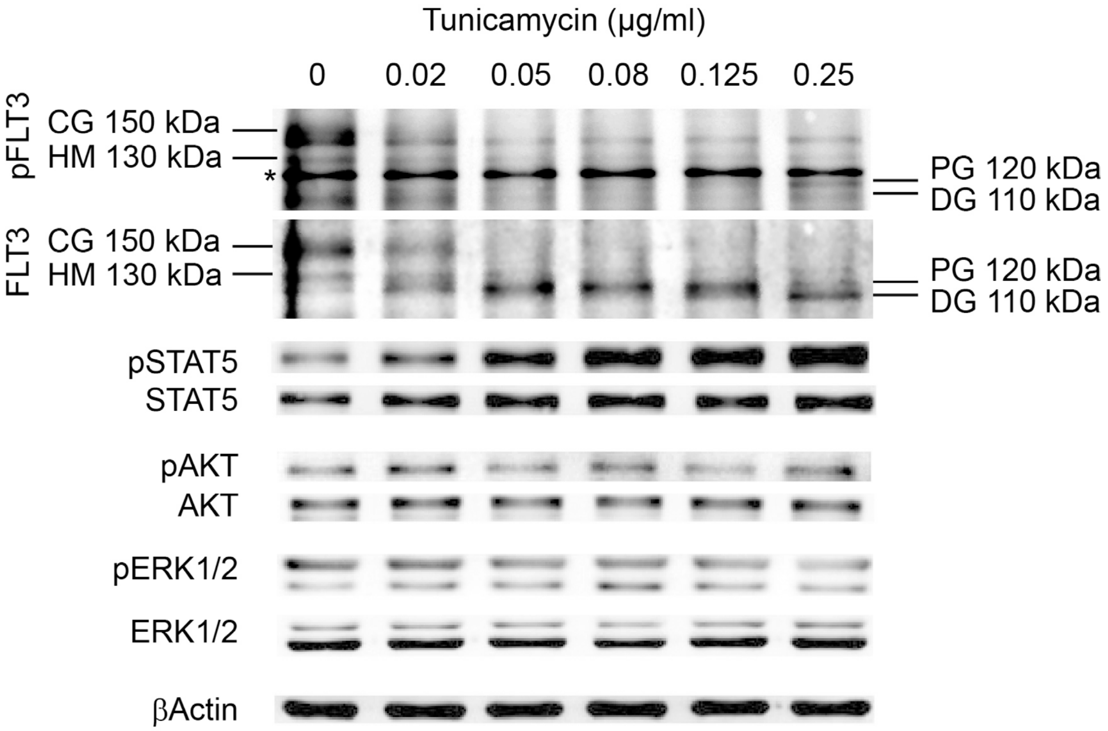

Supplementary Figure 1: Tunicamycin inhibits glycosylation and alters signal transduction of FLT3 ITD. MOLM13 cells harboring endogenous FLT3ITD were treated with the indicated doses of tunicamycin for 24 h. Whole cell lysates were prepared and lysate aliquots were subjected to SDS-PAGE and immunoblotting with the indicated antibodies. CG, complex glycosylated form; HM, high-mannose form; PG, partially glycosylated; DG, deglycosylated. *denotes a non-specific band in the pFLT3 blot. Assignments of molecular masses were done by comparison with marker proteins, assignment of the forms was based on these masses and previous observations [1].

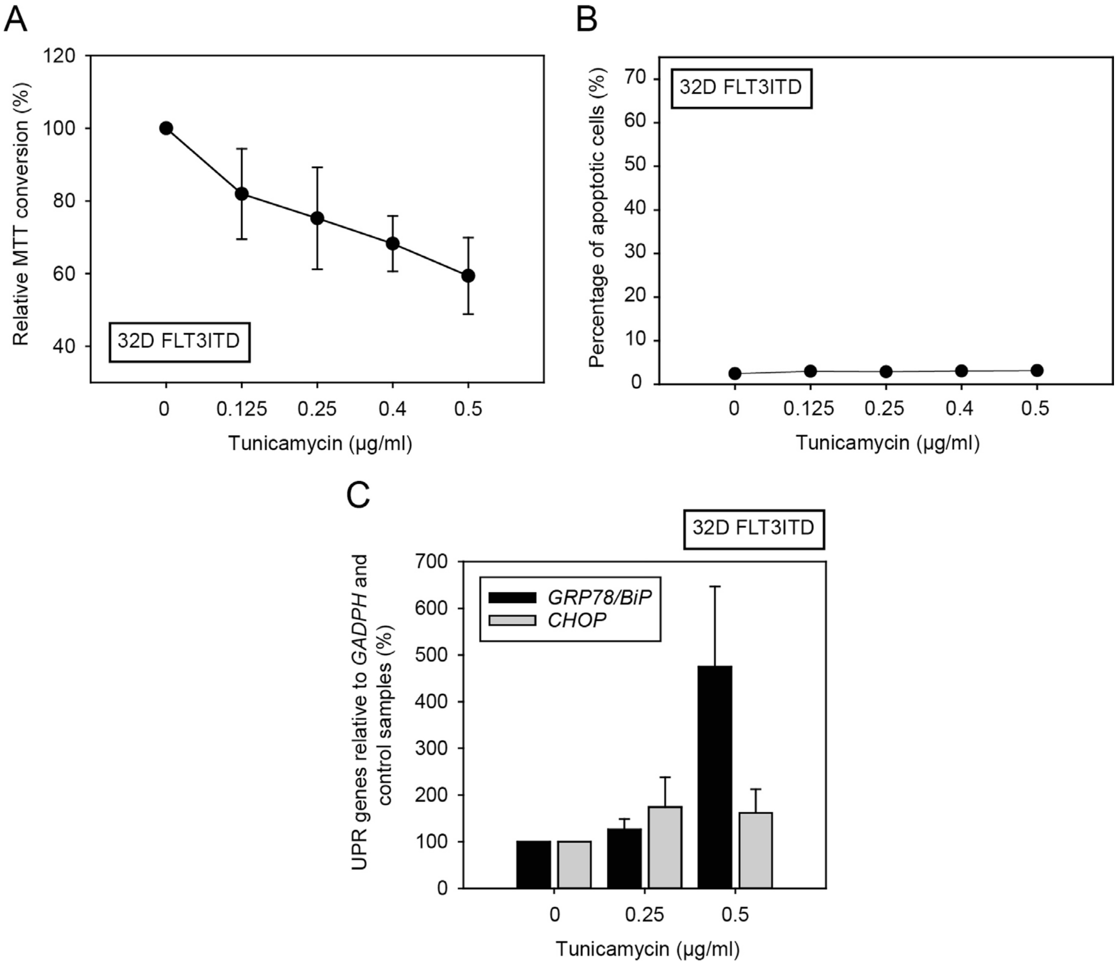

Supplementary Figure 2: Tunicamycin inhibits proliferation, and promotes mRNA expression of unfolded-protein response (UPR) genes in 32D cells expressing FLT3ITD. 32D cells, stably transduced with FLT3ITD, were treated with the indicated concentrations of tunicamycin for 72 h (**A**) or 24 h (**B**, **C**). Subsequently the amount of viable cells was measured by MTT conversion (A), apoptosis was determined using the Annexin V method (B), or RNA was extracted and mRNA expression of *GRP78/BiP* or *CHOP* were determined by RT-qPCR (C). Data are means ± SD; A, *n* = 4; B, *n* = 3; C, *n* = 3.

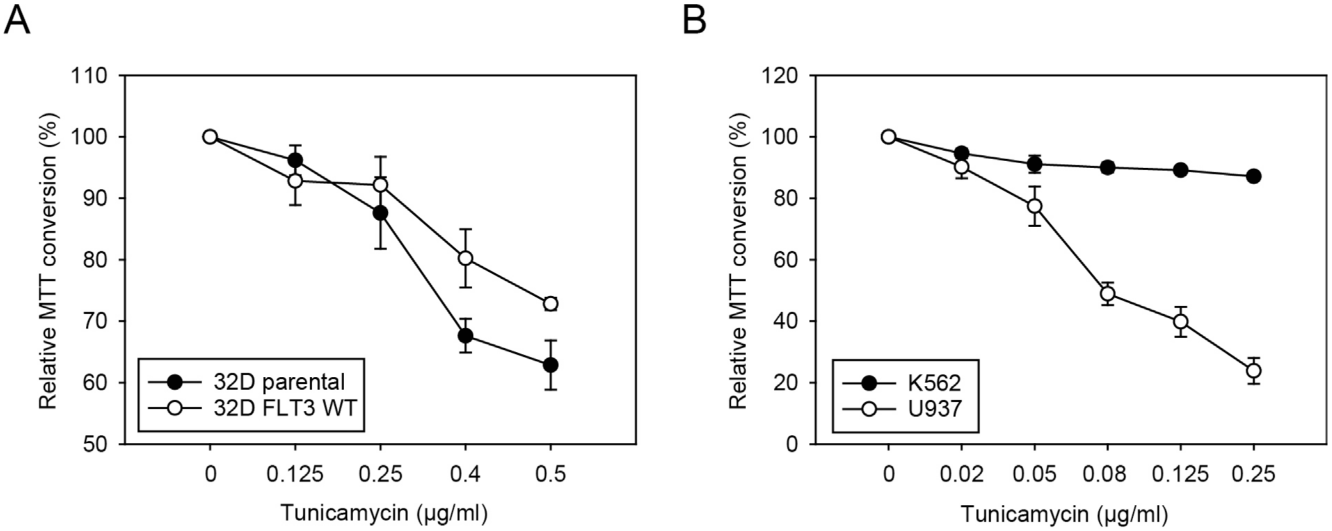

Supplementary Figure 3: Effect of tunicamycin on proliferation and viability of myeloid cells not expressing FLT3ITD. Parental 32D cells, or 32D cells stably transduced with wildtype (WT) FLT3 and cultivated in presence of IL-3 (**A**), or the human myeloid cell lines K562 or U937 (**B**) were treated with the indicated concentrations of tunicamycin for 72 h. Subsequently the amount of viable cells was measured by MTT conversion. Data are means ± SD; *n* = 3.

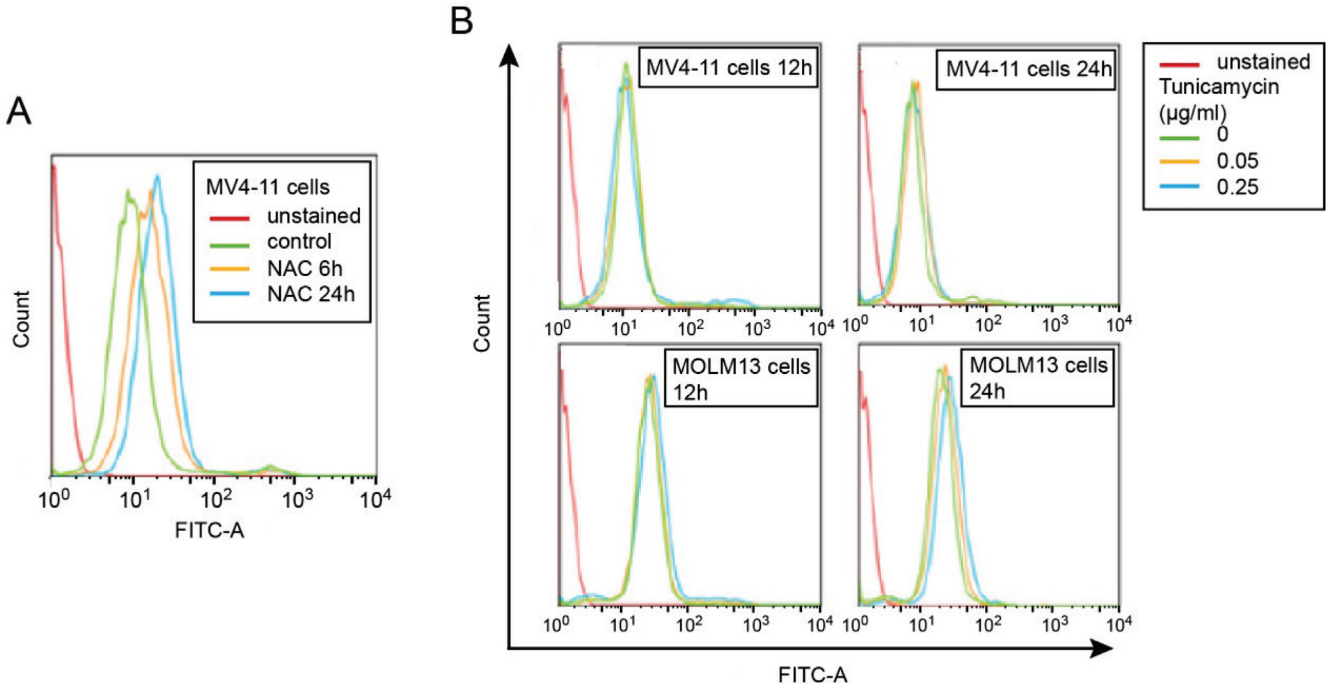

Supplementary Figure 4: ROS formation in MV4-11 and MOLM13 cells is not affected by tunicamycin. (**A**) MV4-11 cells were treated with N-acetylcysteine (1 mM) for the indicated times. Subsequently, cellular ROS levels were measured using the fluorescent dye carboxy-H_2DFFDA as described earlier [2]. Fluorescence was detected with a flow cytometer. Unstained cells served as controls. Note that NAC treatment reduces ROS levels. (**B**) ROS formation in cells pretreated or not with tunicamycin as indicated was monitored as in (A). The shown experiment is representative of three with consistent results.

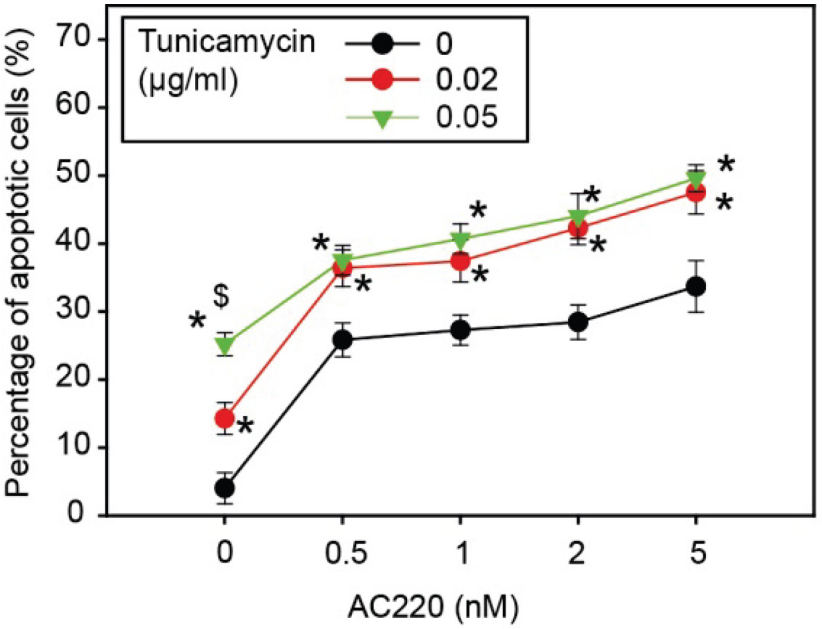

Supplementary Figure 5: Low doses of tunicamycin enhance the pro-apoptotic effect of AC220 in MV4-11 cells. MV4-11 cells were treated simultaneously with the indicated concentrations of AC220 and tunicamycin for 24 h. Subsequently apoptosis was determined using the Annexin V method. Data are means ± SD; $n = 3$; * denotes significant differences compared to controls without tunicamycin; $ significant vs. 0.02 µg/ml tunicamycin.

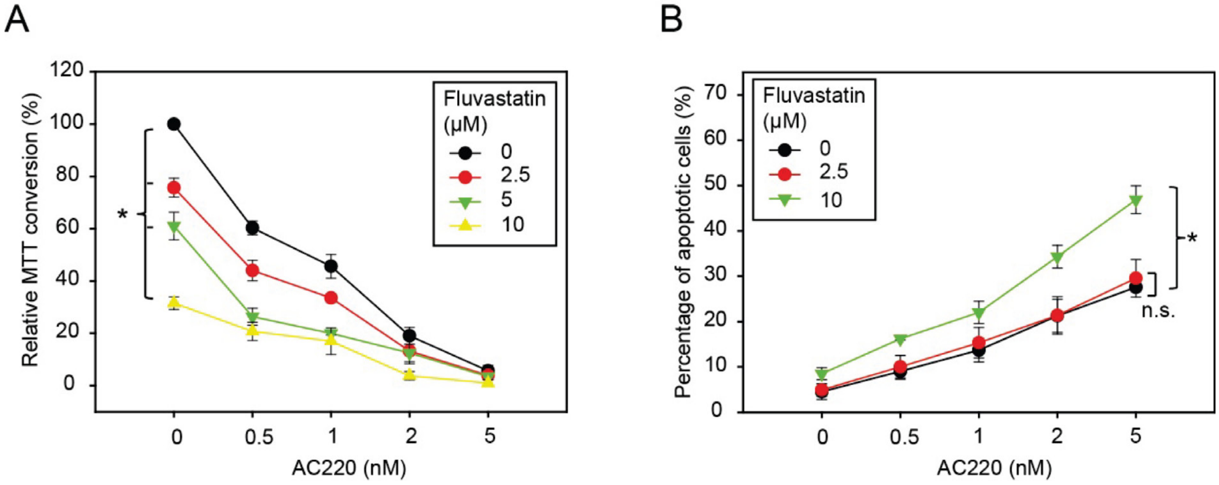

Supplementary Figure 6: Fluvastatin enhances the cytotoxicity of the FLT3 inhibitor AC220. MV4-11 cells were treated simultaneously with the indicated concentrations of AC220 inhibitors and tunicamycin for 72 h (**A**) or 24 h (**B**). Subsequently the amount of viable cells was measured by MTT conversion (A) or apoptosis was determined using the Annexin V method (B). Data are means ± SD; *n* = 3; *denotes significant differences between the treatment series by two-way ANOVA.

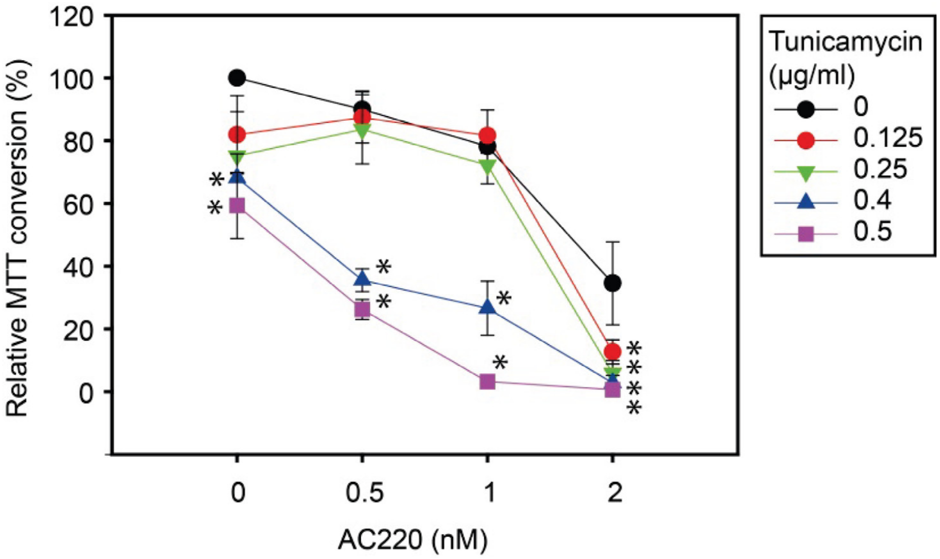

Supplementary Figure 7: Effect of combined treatment with tunicamycin and AC220 on proliferation in 32D cells expressing FLT3ITD. 32D cells, stably transduced with FLT3ITD, were treated with the indicated concentrations of tunicamycin combined with the indicated concentrations of AC220 for 72. Subsequently the amount of viable cells was measured by MTT conversion. Data are means ± SD; *n* = 4; *Treatment conditions under which combination of AC220 with tunicamycin was significantly more effective than treatment with AC220 alone are indicated.

Supplementary Table 1: Tunicamycin synergistically enhances the pro-apoptotic effect of AC220

Compound treatment		MV4-11 cells	MOLM13 cells
**AC220 (nM)**	**Tunicamycin (µg/ml)**	**Annexin V assay (CI)**	
0.5	0.02	**0.254**	
	0.05	**0.538**	
	0.125	**0.467**	**0.681**
1	0.02	**0.264**	
	0.05	**0.466**	
	0.125	**0.496**	1.015
2	0.02	**0.199**	
	0.05	**0.401**	
	0.125	**0.442**	**0.592**
5	0.02	**0.155**	
	0.05	**0.312**	
	0.125	**0.409**	1.058

MV4-11 cells or MOLM13 cells were treated simultaneously with the indicated concentrations of AC220 and tunicamycin for 24 h. Subsequently apoptosis was determined using the Annexin V method. Combination indices were determined according to the method of Chou-Talalay [3]. Combinations with synergism (CI < 1) are highlighted grey.

Supplementary Table 2: The combination of the kinase inhibitor AC220 with tunicamycin synergistically inhibits proliferation of FLT3ITD expressing 32D cells

Compound treatment		32D cells FLT3ITD
**AC220 (nM)**	**Tunicamycin (µg/ml)**	**MTT assay (CI)**
0.5	0.125	2.438
	0.25	2.876
	0.4	**0.429**
	0.5	**0.326**
1	0.125	2.217
	0.25	1.910
	0.4	**0.495**
	0.5	**0.125**
2	0.125	**0.501**
	0.25	**0.331**
	0.4	**0.227**
	0.5	**0.109**

32D cells expressing FLT3ITD were treated simultaneously with the indicated concentrations of AC220 and tunicamycin. Subsequently the amount of viable cells was measured by MTT conversion after 72 h. Combination indices were determined according to the method of Chou-Talalay [3]. Combinations with synergism (CI < 1) are highlighted grey.

REFERENCES

1. Schmidt-Arras DE, Böhmer A, Markova B, Choudhary C, Serve H, Böhmer FD. Tyrosine phosphorylation regulates maturation of receptor tyrosine kinases. Mol Cell Biol. 2005; 25:3690–3703.
2. Godfrey R, Arora D, Bauer R, Stopp S, Müller JP, Heinrich T, Böhmer SA, Dagnell M, Schnetzke U, Scholl S, Östman A, Böhmer FD. Cell transformation by FLT3 ITD in acute myeloid leukemia involves oxidative inactivation of the tumor suppressor protein-tyrosine phosphatase DEP-1/PTPRJ. Blood. 2012; 119:4499–4511.
3. Chou TC. Drug combination studies and their synergy quantification using the Chou-Talalay method. Cancer Res. 2010; 70:440–446.
